# Associations of the Xingfa gamification framework with students' sport motivation and physical fitness: a cross-sectional comparison across 14 schools

**DOI:** 10.3389/fpsyg.2026.1904485

**Published:** 2026-08-27

**Authors:** Ye-Jun Li, Dan Zhu, Xiang-Li Tang

**Affiliations:** 1 Department of Physical Education and Research, Hunan Institute of Technology, Hengyang, China; 2 Digital Research Institute, China National Academy of Educational Sciences, Beijing, China; 3 College of Physical Education, Hunan Normal University, Changsha, China; 4 College of Finance and Economics, Hunan University of Technology and Business, Changsha, China

**Keywords:** gamification, motivation internalization, Physical Education (PE), psychological needs, Self-Determination Theory (SDT)

## Abstract

**Introduction:**

Traditional gamification in physical education (PE) often creates a needs gap. This study examines the associations of Xingfa Teaching Of Physical Education (XFTPE) with students' internalized motivation and physical fitness. It is a structured, three-stage progression aligned with Self-Determination Theory.

**Methods:**

A cross-sectional, comparative design was employed, involving 1,400 primary school students (grades 4–6) from 14 schools. Seven schools implemented XFTPE, while seven used traditional PE. Participants completed the Sport Motivation Scale (SMS-6) and the National Physical Fitness Test. Data were analyzed using Linear Mixed-Effects Models and Pearson correlation.

**Results:**

Students in XFTPE schools reported significantly higher levels of internalized sport motivation (*M* = 5.01, SD = 0.71) compared to those in traditional schools (*M* = 4.63, SD = 0.67) (*B* = 0.37, *P* < 0.001, *d* = 0.26). Internalized motivation was positively correlated with physical fitness scores (*r* = 0.595, *P* < 0.001). Additionally, XFTPE students exhibited higher physical fitness scores (*B* = 1.44, *P* = 0.005), although the effect size was small (*d* = 0.07).

**Discussion:**

These findings indicate an association between the XFTPE approach and higher internalized motivation. The difference in effect sizes between motivational and fitness outcomes underscores the distinction between psychological correlates and physiological adaptation, highlighting the necessity for longitudinal designs in future research.

## 1 Introduction

A paradox exists in contemporary physical education (PE): students often exhibit a natural inclination toward physical games, yet they dislike school-mandated PE classes. The root cause lies in the failure of these classes to satisfy students' basic psychological needs. Traditional PE environments are typically dominated by rigid skill training and instructional content, which impede the development of students' autonomy and competence, thereby diminishing their intrinsic motivation to participate in physical activities (Kantanista and Borowiec, 2021; Ferriz-Valero et al., 2020). Consequently, the primary challenge for PE practitioners today is not merely fulfilling instructional objectives, but rather determining how to cultivate students' genuine enjoyment of PE classes.

To address insufficient student engagement, gamification has been widely advocated as a key strategy to enhance the appeal of physical education (PE) (Chiva-Bartoll and Fernández-Rio, 2021; Arufe-Giráldez et al., 2022). However, current implementations present a problem: they rely excessively on hedonic entertainment or extrinsic rewards, failing to align with deeper psychological drives. Research indicates that while systematically restructuring instructional elements through gamification is associated with significantly improves student enthusiasm and performance (Sotos-Martínez et al., 2024; Quintas et al., 2020; Sotos-Martínez et al., 2023), excessively rich game experiences or an overemphasis on pleasure can undermine deep learning motivation (Fernandez-Rio, 2020; Acharibasam and McVittie, 2023). Self-Determination Theory (SDT) posits that quality engagement in PE relies on fulfilling basic psychological needs, specifically autonomy, competence, and relatedness, rather than merely pursuing hedonic experiences (Zhai et al., 2025; Roure and Pasco, 2022; Chen and Wang, 2017). Therefore, effective gamified PE instruction must transcend entertainment, employing systematic design to align game mechanisms with the fulfillment of these three core SDT needs (Bilton, 2020).

Xingfa Teaching in Physical Education (XFTPE) utilizes a progressive gamification framework to align with SDT psychological needs, aiming to enhance the appeal of PE classes. Compared to standard gamified instruction, XFTPE emphasizes conducting games in natural settings, which is hypothesized to facilitate student psychological readiness and class engagement (Brown, 2017). Furthermore, it designs instruction based on students' motivational states rather than adhering to simplistic game formats: in the initial phase, simple games lower the participation threshold and protect student autonomy in non-controlling environments, such as encouraging spontaneous play during breaks; In the intermediate phase, moderate challenges stimulate curiosity and provide an optimal challenge level to satisfy competence; In the final phase, group games in natural settings foster peer connections to fulfill relatedness (Bernacki and Walkington, 2018; Gopinath Bharathi et al., 2016). By leveraging outdoor environments to trigger situational interest (Ruiz-Navas et al., 2024; Fernández Galeote et al., 2021), XFTPE aims to internalize external game stimuli into intrinsic motivation.

To empirically examine this proposed model, this study employed a comparative survey design examining 1,400 primary school students from XFTPE and traditional schools. Moving beyond mere performance comparisons, this research investigates the central premise of the Xingfa Framework: whether a theory-driven, three-stage gamification design is associated with higher levels of internalized motivation compared to traditional instruction. Furthermore, by examining the motivation-performance nexus, this study explores the association between this structured need-supportive approach and tangible physical outcomes, ultimately discussing its theoretical implications for promoting long-term behavioral engagement in PE. This investigation is informed by recent evidence indicating that while motivation and autonomy support are critical modifiable determinants within school settings, changes in these individual or interpersonal factors do not necessarily translate into changes in physical activity behavior (Khudair et al., 2025; Ling et al., 2024). This complexity necessitates a cautious interpretation of the motivation-fitness association and highlights the importance of objectively measuring both the proposed psychological mechanisms and subsequent behavioral outcomes.

## 2 Background

**Figure 1:**
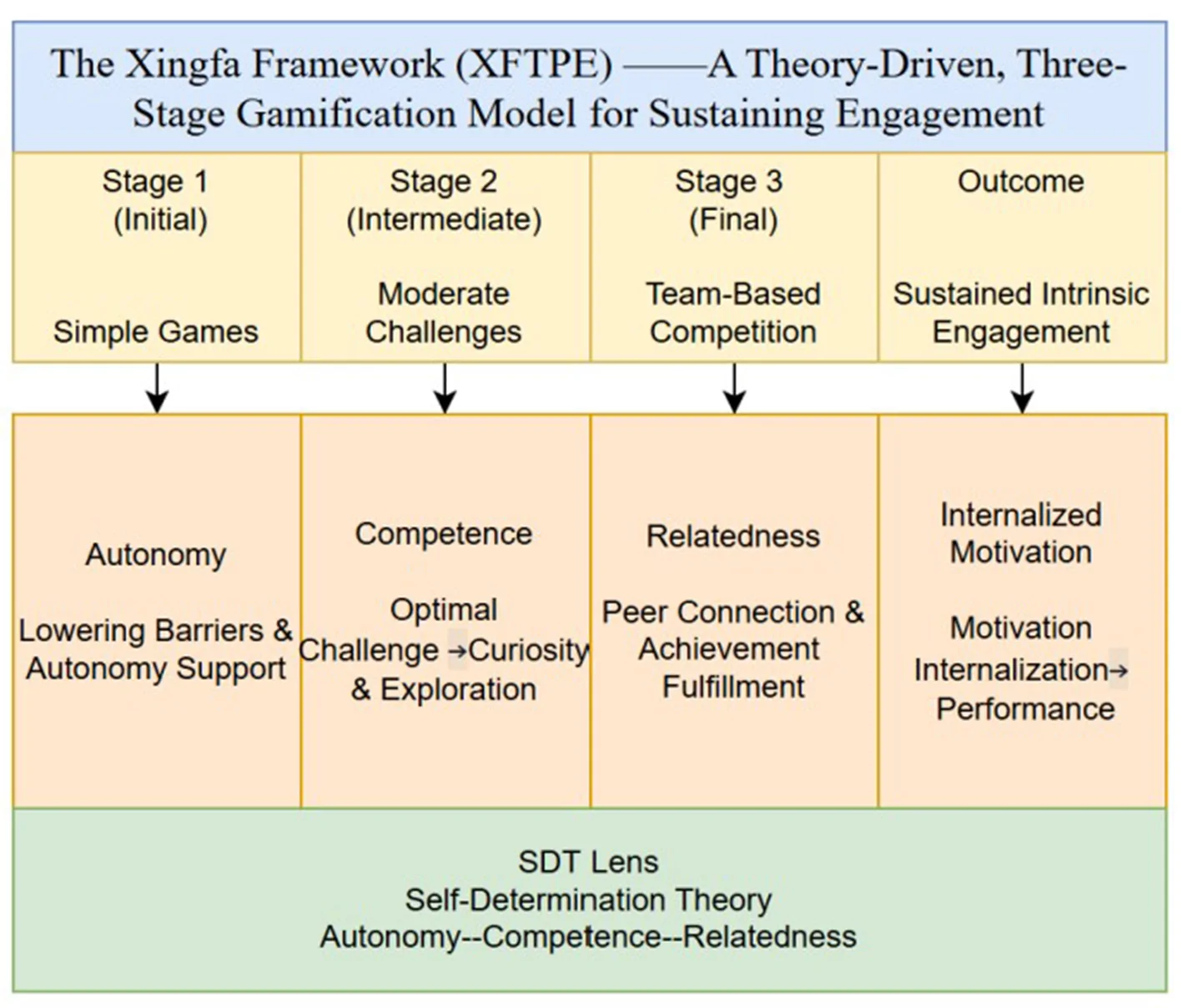
The Xingfa Framework (XFTPE)—a theory-driven, three-stage gamification model for promoting engagement.

The theoretical model in Figure 1 illustrates the XFTPE approach as a structured, three-stage progression designed to cultivate intrinsic motivation by satisfying three basic psychological needs outlined by Self-Determination Theory (SDT): autonomy, competence, and relatedness. The model proposes that the initial stage, utilizing simple games, lowers participation barriers and fosters autonomy. The intermediate stage introduces moderate challenges that stimulate curiosity and exploration, thereby potentially enhancing competence. The final stage incorporates team-based activities that leverage peer interaction to fulfill relatedness. The culmination of this sequential need-supportive process is sustained intrinsic engagement, which theoretically contributes to motivation internalization and is posited to be associated with improved physical performance.

### 2.1 The traditional gamification needs gap

Although gamification incorporates game elements into PE instruction, it typically manifests as isolated, fragmented activities appended to skill-training-dominated curricula (Arufe-Giráldez and Navarro-Patón, 2021). From a Self-Determination Theory (SDT) perspective, this approach creates a “needs gap.” If a gamified activity solely aims to provide unstructured fun and is immediately followed by strict, teacher-led skill training, it may suppress autonomy and potentially induce feelings of incompetence due to high failure rates, thereby negating its psychological benefits. Cognitive Evaluation Theory (CET) specifies that intrinsic motivation is significantly undermined when the learning environment abruptly shifts between non-controlling play and highly controlling instruction (Qiao et al., 2022; Schöbel et al., 2020). Therefore, the ineffectiveness of traditional gamification may be attributed to an instructional design lacking a coherent mechanism to continuously support the three basic psychological needs throughout the lesson.

### 2.2 Motivation internalization mechanism in XFTPE

The theoretical advantage of XFTPE lies in its capacity to facilitate motivation internalization, the process of transforming externally prompted behaviors into self-endorsed behaviors. Organismic Integration Theory (OIT), a core sub-theory of Self-Determination Theory (SDT), explains this process: behaviors initially driven by external cues can only be internalized when the surrounding social context supports individual psychological needs (Chee and Wong, 2017). As illustrated in Figure 1, XFTPE creates this continuous need-supportive environment. By employing progressively intensive games, it ensures that as students' initial curiosity wanes, they are immediately re-engaged by more challenging games, followed by peer cooperative games in natural settings to satisfy relatedness (Deci and Ryan, 2000; Gilal et al., 2018). Within this framework, nature functions as a psychological catalyst; natural environments may inherently reduce students' perceptions of teacher control and spatial constraints, thereby potentially enhancing their autonomy and peer connection (Vansteenkiste et al., 2020; Reeve, 2023). This structured progression is hypothesized to mitigate the motivational decline typical in PE instruction, effectively converting students' transient external engagement into stable intrinsic motivation (Gilal et al., 2020).

### 2.3 From intrinsic motivation to behavioral outcomes

In physical education pedagogy, the ultimate value of internalized motivation lies in its capacity to be associated with sustained physical engagement and improve motor performance. Research emphasizes that when motivation stems from intrinsic drives rather than extrinsic contingencies, students tend to exhibit greater perseverance and superior emotional regulation when facing challenging tasks, ultimately demonstrating higher levels of physical literacy (Hagger and Hamilton, 2020; Koestner et al., 1996). Because the XFTPE framework is specifically designed to cultivate this deep intrinsic motivation by systematically satisfying psychological needs, students in this environment would theoretically be expected to exhibit both higher-quality motivation and better physical fitness outcomes. Therefore, guided by the theoretical mechanisms of need satisfaction and motivational internalization outlined above, this study seeks to address two interrelated questions. First, to what extent does the progressive, three-stage need-supportive environment of XFTPE differ from traditional PE contexts in terms of student motivation? Second, does this theoretically driven motivation positively relate to tangible physical fitness outcomes, and do students in XFTPE schools demonstrate differences in physical performance? Grounded in Self-Determination Theory, we formulated the following hypotheses: *H1*: Students in XFTPE schools will report significantly higher levels of internalized sport motivation compared to students in traditional PE settings. *H2*: Internalized sport motivation will be positively associated with students' physical fitness performance. *H3*: Students in XFTPE schools will demonstrate superior physical fitness outcomes compared to their counterparts in traditional schools.

## 3 Method

### 3.1 Research design and participants

This study employed a cross-sectional, comparative design to investigate the effects of the XFTPE model. The sample was drawn from 14 primary schools (grades 4–6) via cluster sampling, with 7 schools implementing the XFTPE curriculum and 7 matched schools using traditional PE. A total of 1,400 valid student responses were collected. The 14 participating schools were located across three administrative regions: Changsha city, Hengyang city, and Shenzhen City. Selection was based on a multi-stage stratified cluster sampling approach. To control for regional and demographic differences, schools were first stratified by region. Within each region, schools were purposefully selected based on administrative feasibility and the willingness of school principals to participate. To minimize selection bias, experimental and control schools were matched at the regional level. Specifically, for each XFTPE school in a given region, a comparison school from the same region was selected based on the following matching criteria: (a) similar school size (approximate number of students in grades 4–6), (b) comparable socioeconomic status of the school district (determined by the district education bureau's classification), and (c) equivalent PE facilities and resources. This within-region matching process was conducted to control for potential regional differences in educational policies, climate, and cultural attitudes toward PE. Regarding group allocation, assignment to the XFTPE curriculum was predetermined. The seven experimental schools had voluntarily adopted and implemented the Xingfa Framework as part of a pilot program for at least one academic year prior to data collection. The seven comparison schools were selected from those maintaining standard PE instruction without any formal gamification intervention. Although random assignment was not feasible due to practical constraints, baseline equivalence was assessed. Independent samples *t*-tests comparing the average BMI and previous year's fitness test scores of the two groups showed no statistically significant differences at baseline (*P* > 0.05), suggesting that the groups were largely comparable at the onset of the study.

The demographic characteristics of the participants were as follows: The mean age of the participants was 10.98 years (SD = 0.89), ranging from 10 to 12 years. The sample consisted of 713 boys (50.9%) and 687 girls (49.1%). Regarding inclusion criteria, students were eligible if they were enrolled in grades 4–6, attended PE classes during the data collection period, and provided written informed consent. Students were excluded if they had medical exemptions from physical activity, physical disabilities preventing standard fitness testing, or incomplete data records. Detailed demographic breakdowns by group are presented in Table 1. And the research was conducted in accordance with the Declaration of Helsinki and approved by the Biomedical Research Ethics Committee of Hunan Normal University; with license number 772. Data were collected between November 2024 and July 2025.

**Table 1:** Demographic characteristics of participants by group.

Variable	Total (*N* = 1400)	XFTPE group (*n* = 700)	Traditional group (*n* = 700)
Age (years)	10.98 ± 0.89	10.91 ± 0.83	11.05 ± 0.94
Gender, *n* (%)			
Boys	713 (50.9%)	355 (49.8%)	358 (50.2%)
Girls	687 (49.1%)	345 (50.2%)	342 (49.8%)
Grade, *n* (%)			
Grade 4	497 (35.5%)	278 (55.9%)	219 (44.1%)
Grade 5	452 (32.3%)	229 (50.7%)	223 (49.3%)
Grade 6	451 (33.2%)	193 (42.8%)	258 (57.2%)

### 3.2 Measures

#### 3.2.1 The sport motivation scale

The Sport motivation Scale-6 (SMS-6) was used to measure students' sports motivation (Mallett et al., 2006). This version is an optimized version of the Sport motivation scale (SMS) (Pelletier et al., 1995). According to the development stage of children, the Chinese version of the Sport motivation Scale was referred to, and the terms of the items were precisely defined to conform to the cognitive understanding of primary school students. This scale contains a total of 24 items, among which there are 4 reverse-coded items. These items had a closed response option, following a Likert scale from 1 to 7, with 1 (totally disagree) to 7 (totally agree). This scale mainly includes the following six dimensions: Amotivation (Q5, Q12, Q17, Q22); External Regulation (Q4, Q11, Q19, Q24); Introjected Regulation (Q7, Q10, Q16, Q23); Identified Regulation (Q3, Q8, Q15, Q20); Integrated Regulation (Q2, Q9, Q13,Q21); Intrinsic Motivation (Q1, Q6, Q14, Q18).

Prior to the formal investigation, this study randomly selected 300 primary school students and distributed the Sports Motivation Scale to them in order to test the reliability and validity of the scale. The results are shown in Table 2. Cronbach's α was used to evaluate the internal consistency of the questionnaire (Stensen, 2022), the results revealed that the Cronbach's α of the scale was 0.886, indicating good reliability. Each item was also subjected to testing, and the Corrected Item-Total Correlation (CITC) between the observed variables and their latent variables was reported. Each item must meet the criterion of being greater than 0.40 to demonstrate that the measurement items possess good fit and fulfill the requirements (Hobart, 2009). Based on the results presented in Table 2, five items, namely Q4, Q11, Q23, Q2, and Q6, were deleted.

**Table 2:** Reliability of the sport motivation scale.

Variate	Item	CITC	Cronbach's alpha if item deleted	Cronbach's alpha
Sport motivation	—	—	—	0.886
Amotivation	Q5	0.477	0.881	0.878
	Q12	0.504	0.881	
	Q17	0.462	0.881	
	Q22	0.499	0.880	
External regulation	Q4	0.246	0.888	0.878
	Q11	0.387	0.883	
	Q19	0.470	0.881	
	Q24	0.458	0.882	
Introjected regulation	Q7	0.486	0.881	0.861
	Q10	0.546	0.879	
	Q16	0.539	0.879	
	Q23	0.395	0.883	
Identified regulation	Q3	0.555	0.879	0.859
	Q8	0.510	0.880	
	Q15	0.575	0.878	
	Q20	0.524	0.880	
Integrated regulation	Q2	0.363	0.884	0.864
	Q9	0.441	0.882	
	Q13	0.548	0.879	
	Q21	0.489	0.881	
Intrinsic motivation	Q1	0.441	0.882	0.866
	Q6	0.266	0.887	
	Q14	0.548	0.879	
	Q18	0.530	0.879	

**Figure 2:**
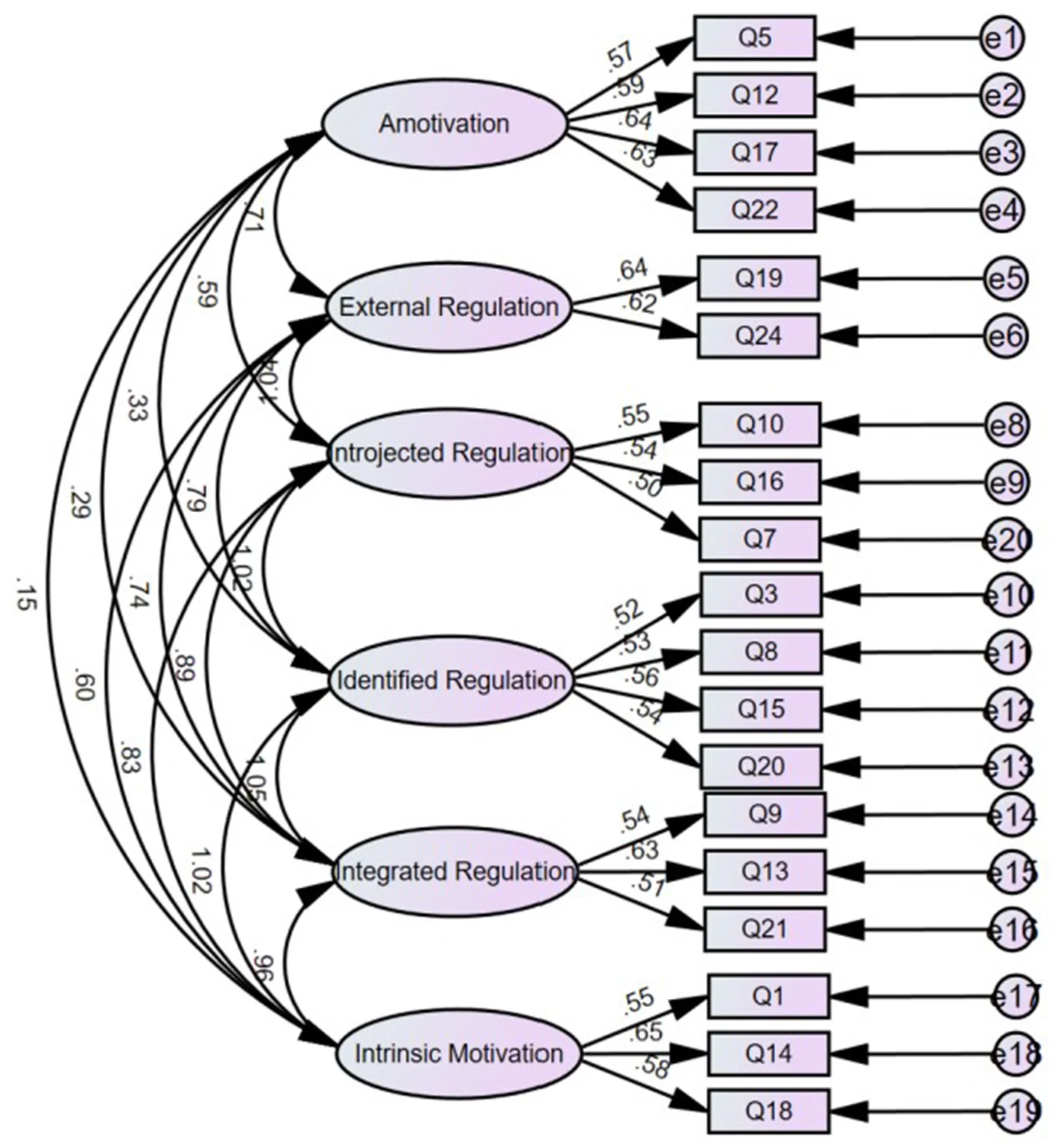
CFA model of sport motivation.

On the basis of satisfactory reliability testing, a confirmatory factor analysis (CFA) was conducted. Figure 2 illustrates the confirmatory CFA model for the Sport Motivation Scale. The model fit indices are as follows: CMIN/DF = 4.425; GFI = 0.946; AGFI = 0.925; IFI = 0.913; CFI = 0.913; TLI = 0.891 (see Table 3). The data indicate that the overall goodness-of-fit indices of the scale generally meet the criteria, suggesting that the scale demonstrates relatively reasonable validity.

**Table 3:** The model fit of the sport motivation scale.

Scale	CMIN/DF	GFI	AGFI	IFI	CFI	TLI
Sport motivation	4.425	0.946	0.925	0.913	0.913	0.891

Finally, composite reliability (CR) and convergent validity (AVE) tests were conducted on the scale, with the results presented in Table 4. Composite reliability (CR) measures the extent to which each latent variable can be consistently explained by all the measurement items that constitute it. A CR value greater than 0.7 indicates good composite reliability, while a value above 0.6 is considered acceptable. An average variance extracted (AVE) value exceeding 0.50 suggests favorable convergent validity, whereas values ranging from 0.36 to 0.5 are deemed acceptable (Zhu et al., 2025). The evaluation of AVE and CR values should be made in conjunction with model fit indices. In summary, the validity of the scale is deemed acceptable. The scale ultimately consists of 19 items across six dimensions, specifically: Amotivation (Q5, Q12, Q17, Q22); External Regulation (Q19, Q24); Introjected Regulation (Q7, Q10, Q16); Identified Regulation (Q3, Q8, Q15, Q20); Integrated Regulation (Q9, Q13, Q21); and Intrinsic Motivation (Q1, Q14, Q18).

**Table 4:** Results of the convergent validity of the sport motivation scale.

Variate	Item	Standardized estimate	CR	AVE	*P*
Amotivation	Q5	0.574	0.704	0.473	
	Q12	0.593			—
	Q17	0.642			< 0.001
	Q22	0.634			< 0.001
External regulation	Q19	0.643	0.609	0.400	—
	Q24	0.622			< 0.001
Introjected regulation	Q7	0.504	0.544	0.385	—
	Q10	0.553			< 0.001
	Q16	0.544			< 0.001
Identified regulation	Q3	0.524	0.623	0.393	—
	Q8	0.533			< 0.001
	Q15	0.564			< 0.001
	Q20	0.544			< 0.001
Integrated regulation	Q9	0.543	0.583	0.360	—
	Q13	0.634			< 0.001
	Q21	0.514			< 0.001
Intrinsic motivation	Q1	0.553	0.624	0.368	—
	Q14	0.654			< 0.001
	Q18	0.584			< 0.001

#### 3.2.2 The national physical fitness test for primary school students

The National Student Physical Fitness and Health Standard serves as an evaluative criterion for assessing students' physical health status and the effectiveness of physical exercise. Issued by the Ministry of Education of China, it outlines the fundamental requirements for students' physical health across different age groups and provides a basis for evaluating individual students' physical health conditions. The physical fitness test results are evaluated using standardized performance metrics derived from the Chinese National Physical Fitness Test, rather than direct physiological measurements expressed in International System of Units (SI). The test items are divided into three major categories: (a) Body shape category: height, weight; (b) Physical function category: vital capacity; (c) Physical fitness category: 50-m run, sit-and-reach, 1-min rope skipping, 1-min sit-ups, 50 m × 8 shuttle run. For instance, the physical fitness test content for fifth-grade students comprises BMI, Vital Capacity, 50-m run, Sit-and-Reach, 1-min Rope Skipping, 1-min Sit-ups, and 50 m × 8 Shuttle Run. The maximum scores for each item are 15 points, 15 points, 20 points, 10 points, 10 points, 20 points, and 10 points, respectively. The total score is out of 100 points, with classifications as follows: 90.0 points or above for “Excellent,” 80.0 to 89.9 points for “Good,” 60.0 to 79.9 points for “Pass,” and 59.9 points or below for “Fail.” The specific indicators and their weights are detailed in Table 5. Scoring for each item is based on actual test data, following predefined criteria to ensure objective evaluation, the specific scoring criteria for each project can be found in the Appendix I.

**Table 5:** Individual indicators and weights.

Test object	Single indicator	Weight
Grade one to grade six	Body Mass Index(BMI)	15
	Vital Capacity	15
Grades three and four	50-m run	20
	sit-and-reach	20
	1-min rope skipping	20
	1-min sit-ups	10
Grades five and six	50-m run	20
	sit-and-reach	10
	1-min rope skipping	10
	1-min sit-ups	20
	50 m × 8 shuttle run	10

#### 3.2.3 Comparison between Xingfa teaching and traditional teaching

Comparison between Xingfa Teaching and Traditional Teaching. Specifically, the Xingfa teaching of Physical Education (XFTPE) advocates for conducting approximately one 1 h of sports game activities per day. During this period, there is not an excessive emphasis on the acquisition of sports skills; rather, it stresses the experience of nature and play within a created game atmosphere. Teaching is then carried out when students show interest in the game content. A comparison of the intended design characteristics between the Xingfa classes and traditional PE classes is presented in the following Table 6. The descriptions outline the theoretical framework and planned structural elements of the respective approaches. Among these, the traditional PE classes listed emphasize a universal phenomenon.

**Table 6:** Comparative table of Xingfa teaching and traditional teaching.

Aspect	Xingfa classes	Traditional PE classes
Sessions per week	5	3
Weekly class hours	Usually 4–5 h	Generally 3-4 hours
Typical activity content	In gamified team games, there are clear rules and objectives. For instance, in a “sports relay race with a twist,” students need to complete a series of physical tasks (e.g., jumping over hurdles, throwing a ball into a target) before passing the baton to the next team member. The nature exploration activities involve learning about local flora and fauna while engaging in physical movements like crouching to observe insects or balancing on logs.	Skill drills are highly structured. For example, in a basketball skill drill, students may be required to practice dribbling around cones in a specific time frame. Sports matches follow standard rules of the respective sports, with a focus on competition and skill application.
Progression schemes	Progressive challenge levels: Students start with simple gamified activities and gradually move on to more complex ones. For example, in a team-building game, they may first play a basic cooperation game and then progress to a more challenging one that requires advanced strategic thinking and physical coordination. In nature exploration, they may begin with short hikes on easy trails and progressively take on longer, more challenging routes that demand greater outdoor knowledge and practical skills.	Linear skill progression: Students learn and master basic sports skills first, such as the fundamental movements in gymnastics. Once they have achieved a certain level of proficiency, they move on to more advanced skills in a sequential manner. For example, in swimming, they start with floating and then progress to different swimming strokes.
Progression arrangement plan	At the beginning of the semester, conduct an assessment of students' physical abilities and interests. Based on the results, divide students into different groups with similar levels. Start with basic gamified activities suitable for all groups. As the semester progresses, gradually increase the difficulty of the games and nature exploration activities according to the students' progress. Provide regular feedback and adjust the progression plan as needed.	Based on the grade level, relevant sports skills or theoretical knowledge of health care were uniformly arranged for learning.
Teacher training elements	Specialized training in gamification techniques, including how to design engaging gamified sports activities. Knowledge of affective learning theories to understand and respond to students' emotional needs during physical activities. Experience in outdoor education and nature-related activities.	Traditional PE methodology training, covering the teaching of basic sports skills, rules of various sports, and safety protocols. Familiarity with standard sports testing and evaluation methods (Krath and Schürmann, 2021).
Teaching principles	Satisfy students' interests.	To teach a certain skill or knowledge.

#### 3.2.4 Differentiation from traditional gamification approaches

Beyond distinguishing XFTPE from traditional PE instruction, it is crucial to clarify its novelty relative to previously published gamification approaches in physical education. While gamification is widely advocated to enhance engagement, current implementations often suffer from two limitations that XFTPE specifically addresses.

First, regarding the integration mechanism, traditional gamification in PE typically manifests as isolated, fragmented activities appended to skill-training-dominated curricula (Krath and Schürmann, 2021). This approach creates a needs gap; if a gamified activity solely aims to provide unstructured fun and is immediately followed by strict, teacher-led skill training, it may suppress autonomy and induce feelings of incompetence due to high failure rates. In contrast, XFTPE employs a structured, three-stage progression. This design ensures a continuous need-supportive environment where game mechanics are not merely add-ons but are systematically aligned with the sequential satisfaction of autonomy, competence, and relatedness throughout the lesson.

Second, regarding the motivational mechanism, many existing gamification models rely heavily on extrinsic rewards or hedonic entertainment to trigger short-term behavioral compliance (Arufe-Giráldez et al., 2022; Kurni and Krishnamaneni, 2026). Conversely, XFTPE is uniquely designed to facilitate motivation internalization. Drawing on Organismic Integration Theory (OIT), the framework leverages progressive challenges and peer cooperation in natural settings to transform external engagement into self-endorsed intrinsic motivation, rather than fostering dependency on external incentives.

Finally, XFTPE distinguishes itself by leveraging natural environments as a psychological catalyst, a feature often absent in digital or indoor-based gamification. This non-digital, nature-integrated approach offers a scalable and equitable solution for schools, positioning XFTPE as a shift from gamification as entertainment to gamification as need-supportive design.

### 3.3 Xingfa framework (XFTPE) intervention

This section provides a detailed description of the Xingfa Framework (XFTPE) intervention to facilitate replication.

#### 3.3.1 Lesson structure and duration

The XFTPE intervention was implemented as a structured, three-stage gamification model, designed to progressively satisfy students' psychological needs for autonomy, competence, and relatedness. The model follows a “Play-Practice-Apply” logic, integrated into a typical 45-min PE class period with the following approximate time allocations:

Stage 1: Autonomy-Fostering (10 min), Initial Xing (Desire Arousal): The session began with simple, low-threshold games or warm-up activities in natural settings (e.g., school gardens or playgrounds). These activities were designed to be non-controlling and open-ended, encouraging spontaneous play and exploration. The primary goal was to reduce perceived teacher control, awaken students' initial desire for movement, and foster a sense of autonomy.

Stage 2: Competence-Enhancing (20 min), Middle Xing (Skill Internalization): This stage constituted the core instructional component of the lesson. Teachers guided students through the learning and practice of fundamental motor skills or specific sports skills (e.g., basketball dribbling, track and field basics). Instruction was designed to be gamified with moderate challenges that matched students' skill levels, aiming to satisfy their need for competence through mastery experiences.

Stage 3: Relatedness-Supporting (15 min), Final Xing (Application in Nature): The session concluded with team-based competitive games or application activities conducted in natural environments. In this stage, students utilized the skills learned in Stage 2 within a game context. These activities required cooperation, communication, and strategic thinking, fostering peer connections and a sense of belonging through shared experiences in nature.

#### 3.3.2 Implementation procedures

XFTPE was implemented by trained PE teachers who had undergone a 30-day specialized training program. The training covered the theoretical foundations of SDT, the three-stage progression of XFTPE, and practical guidance on transitioning from skill practice to game application. Teachers were provided with a detailed lesson plan template and a handbook of activity examples. During the study period, teachers participated in monthly peer supervision meetings to discuss implementation challenges and share best practices, ensuring fidelity to the XFTPE model.

#### 3.3.3 Teacher preparation

To ensure the integrity of the XFTPE intervention, several fidelity monitoring procedures were implemented:

Teacher Self-Report: teachers completed a brief, weekly fidelity checklist to report their adherence to the three-stage structure.

Peer Observation: a subset of lessons were observed by a member of the research team using a standardized observation protocol. The protocol assessed the presence of key XFTPE elements, such as the progression from skill practice to game application and the use of natural environments.

Documentation Review: teachers maintained a log of each lesson, including a brief description of the activities used and any deviations from the planned structure. These logs were reviewed periodically to ensure consistency. For detailed operational procedures, please refer to Appendix II.

### 3.4 Data analysis

A two-step analytical approach was adopted to address the nested data structure and research questions. First, Intraclass Correlation Coefficients (ICCs) were calculated to assess the degree of clustering at the school level. The negligible ICC values (ICC (1) = 0.0077 for motivation, ICC (1) = 0.0082 for fitness) indicated that the proportion of variance attributable to between-school differences was minimal. Consequently, the assumption of independence was deemed to hold sufficiently, justifying the use of linear mixed-effects models (LMM) as a valid and robust analytical approach to account for the clustered nature of the data. Subsequently, analyses were conducted to directly test the study's research questions. A Linear Mixed-Effects Model (LMM) was employed to compare SMS-6 and physical fitness scores between the XFTPE and traditional groups. To examine the motivation-performance relationship, a Pearson correlation analysis was performed across the total sample, followed by a second independent samples *t*-test to compare the standardized fitness scores between the two groups. All statistical analyses were conducted using SPSS 22.0, with statistical significance set at *P* < 0.05.

## 4 Results

Descriptive statistics and the results of inferential analyses for the primary study variables are summarized in Table 7. The results provided support for all three hypotheses proposed in this study.

**Table 7:** Descriptive statistics and inferential results for internalized motivation and physical fitness across instructional groups.

Variable	Sport motivation		Physical fitness	
Group	XFTPE	Traditional	XFTPE	Traditional
*N*	700	700	700	700
M ± SD	5.01 ± 0.71	4.63 ± 0.67	83.10 ± 9.32	81.39 ± 9.91
*r* (Total)	0.595^*^			
*B*	0.37	(Reference)	1.44	(Reference)
SE	0.04		0.51	
*T*	9.87		2.81	
*D*	0.26		0.07	
*P*	< 0.001		0.005	
95% CI	[0.30, 0.44]		[0.44, 2.44]	

^*^indicates that *P* < 0.05.

Regarding *H1*, which predicted that students in XFTPE schools would report significantly higher levels of internalized sport motivation, a linear mixed-effects model (LMM) revealed a statistically significant association between the instructional approach and student motivation. Students in the schools implementing the Xingfa Framework (XFTPE) reported higher levels of motivation (*M* = 5.01, SD = 0.71) compared to students in schools using traditional physical education (*M* = 4.63, SD = 0.67). The model estimated that XFTPE students scored 0.37 points higher on the motivation scale than their traditional PE counterparts [*B* = 0.37, *SE* = 0.04, *t* = 9.87, *P* < 0.001, 95% CI (0.30, 0.44)]. Thus, *H1* was supported.

Regarding *H2*, which posited a positive association between internalized sport motivation and physical fitness, a Pearson correlation analysis indicated a statistically significant positive relationship across the total sample (*r* = 0.595, *P* < 0.001). This finding aligns with the theoretical premise that intrinsic drives are associated with behavioral persistence. Therefore, *H2* was supported.

Regarding *H3*, which anticipated superior physical fitness outcomes for XFTPE students, the LMM showed a statistically significant difference. XFTPE students demonstrated higher physical fitness scores (*M* = 83.10, SD = 9.32) than students in traditional PE (*M* = 81.39, SD = 9.91). The model estimated that XFTPE students scored 1.44 points higher than traditional PE students [*B* = 1.44, SE = 0.51, *t* = 2.81, *P* =0.005, 95% CI (0.44, 2.44)]. However, the effect size for this difference was small (Cohen's *d* = 0.07). Thus, *H3* was supported, although the practical significance of the difference appears limited.

## 5 Discussion

Previous research attributes the failure of conventional gamification in physical education (PE) to poorly integrated curricula, yet the specific psychological mechanism causing student disengagement remained empirically underexplored (Arufe-Giráldez and Navarro-Patón, 2021). The moderate effect size for motivation associated with the Xingfa Framework (XFTPE) (|d| = 0.26) is consistent with a preliminary indication of its potential. The finding that students in XFTPE schools reported higher motivation is consistent with the theoretical premise that the structured, three-stage approach may be associated with greater need satisfaction compared to traditional instruction. This aligns with the theoretical rationale that a progressive, need-supportive environment could foster more positive psychological states (Gilal et al., 2020).

A key innovation of this study is the empirical examination of XFTPE, a non-digital instructional framework grounded in Self-Determination Theory (SDT). Unlike many digital gamification models that rely on extrinsic rewards and complex technology (Chiva-Bartoll and Fernández-Rio, 2021; Arufe-Giráldez et al., 2022), XFTPE's core strength lies in its structured, three-stage teaching process designed to foster intrinsic motivation and need satisfaction within natural learning environments. This approach offers a scalable and equitable alternative for schools lacking technological resources, addressing a critical barrier in current gamification discourse.

However, it is crucial to situate this study findings within the broader context of intervention research. Although the data were collected from three administrative regions, the generalizability of the findings may still be limited to primary schools within this specific geographical scope. Recent study highlight that many physical activity interventions for youth have shown limited or negligible effects. These research emphasize that individual-level determinants, such as motivation, are important but must be considered within a complex, multi-component framework that also includes interpersonal, institutional, environmental, and policy factors (Ciaccioni et al., 2026; Kolovelonis et al., 2024). This research, while finding an association between XFTPE and motivation, should be interpreted with this complexity in mind. The observed effect on motivation is not an isolated causal pathway but one component within a larger system of influences on student outcomes.

Furthermore, XFTPE distinguishes itself by prioritizing holistic student development as its primary goal, rather than merely focusing on short-term engagement or performance metrics. This aligns with SDT's emphasis on fostering autonomous, competent, and related individuals (Hagger and Hamilton, 2020; Koestner et al., 1996). The moderate effect size for motivation (|d| = 0.26) suggests that XFTPE's theory-driven design may be more effective at fostering internalized motivation compared to approaches that rely heavily on external incentives. While the non-randomized, cross-sectional design prevents definitive causal claims, the observed association between XFTPE and higher motivation is notable. The contextual differences between XFTPE and traditional schools, while introducing complexity, also strengthen the ecological validity of our findings, suggesting that XFTPE can be associated with positive outcomes even when implemented with distinct contextual advantages.

Regarding the fitness outcomes, the small effect size (|d| = 0.07) is consistent with the cross-sectional design, which captures immediate psychological correlates but not the temporal delay required for physiological adaptation. This finding highlights a critical distinction: while XFTPE shows promise for enhancing motivation, translating this psychological shift into measurable fitness improvements likely requires sustained behavioral repetition over time, a pathway that a cross-sectional study cannot fully elucidate. In summary, this study contributes to the literature by demonstrating that a non-digital, theory-driven instructional framework like XFTPE can be associated with positive psychological outcomes in a real-world setting. By focusing on intrinsic motivation, need satisfaction, and holistic development within natural contexts, XFTPE is associated with promising, scalable, and equitable student engagement in PE, distinct from technology-dependent gamification models (Martín-Rodríguez and Madrigal-Cerezo, 2025; Valencia Pilamunga et al., 2026).

Finally, the generalizability of these findings may be constrained by the specific educational and cultural context of China. The XFTPE model was implemented within a system where Physical Education is gaining increasing prominence, yet it still operates within specific structural parameters. Cultural attitudes toward physical activity and the inherent hierarchical nature of teacher-student relationships in China might influence how the need-supportive elements of the framework are perceived and internalized by students (Arsapakdee et al., 2025). Therefore, caution is warranted when attempting to apply these findings to Western educational settings, which may differ significantly in terms of curriculum autonomy, resource allocation, and pedagogical norms. Future research should examine how XFTPE adapts to diverse cultural and systemic environments to establish its cross-cultural validity.

## 6 Conclusion

Through the lens of Self-Determination Theory, this study indicates that the Xingfa Framework (XFTPE) may effectively bridge the gap between superficial entertainment and the fulfillment of deep psychological needs. As a theory-driven, progressive framework, it provides a viable pathway to sustain long-term engagement in physical education. The empirical evidence indicates an association between a structured, three-stage instructional design aligning task complexity with students' shifting psychological states and support for autonomy, competence, and relatedness. While this study reveals a positive association with intrinsic motivation, future research is needed to directly validate the mediating role of psychological need satisfaction. However, the findings also highlight a critical temporal constraint in the motivation-to-performance pathway. While the XFTPE model is associated with the psychological conditions conducive to sustained engagement, translating this shift into measurable physiological adaptation likely requires sustained behavioral repetition over time. This suggests that the potential instructional advantage of need-supportive models may not be fully captured by short-term, cross-sectional fitness metrics. Future research must therefore adopt longitudinal designs to track the delayed, cumulative effects of motivation on physical outcomes. Practically, this study offers a scalable, non-digital solution for schools lacking technological resources. By leveraging natural environments and progressive task structures, XFTPE provides an evidence-based method to transition from fragmented activities to structured need support, potentially promoting both student engagement and educational equity in PE. The model's observed association with intrinsic motivation, as reflected by a moderate effect size, positions it as a valuable tool for advancing the theoretical and practical goals of the field.

## Data Availability

The original contributions presented in the study are included in the article/Supplementary material, further inquiries can be directed to the corresponding author.
