# Supplementary material for: Associations of the Xingfa gamification framework with students' sport motivation and physical fitness: a cross-sectional comparison across 14 schools
Source: Front Psychol. 2026 Aug 27;17:1904485. doi: 10.3389/fpsyg.2026.1904485 (PMC13558109; doi:10.3389/fpsyg.2026.1904485)
Supplement: Supplementary file 1 [file Supplementary_file_1.pdf]

Appendix I

Table 1 Body mass Index scoring criteria(kg/m^2)

Sex	Rating	Sc-ore	Grade One	Grade Two	Grade Three	Grade Four	Grade Five	Grade Six
Male	Normal	100	13.5-18.1	13.7-18.4	13.9-19.4	14.2-20.1	14.4-21.4	14.7-21.8
	Low Weight	80	≤13.4	≤13.6	≤13.8	≤14.1	≤14.3	≤14.6
	Overweight		18.2-20.3	18.5-20.4	19.5-22.1	20.2-22.6	21.5-24.1	21.9-24.5
	Obesity	60	≥20.4	≥20.5	≥22.2	≥22.7	≥24.2	≥24.6
Fem-ale	Normal	100	13.3-17.3	13.5-17.8	13.6-18.6	13.7-19.4	13.8-20.5	14.2-20.8
	Low Weight	80	≤13.2	≤13.4	≤13.5	≤13.6	≤13.7	≤14.1
	Overweight		17.4-19.2	17.9-20.2	18.7-21.1	19.5-22.0	20.6-22.9	20.9-23.6
	Obesity	60	≥19.3	≥20.3	≥21.1	≥22.1	≥23.0	≥23.7

Table 2 Scoring criteria for vital capacity(ml)

Sex	Rating	Score	Grade One	Grade Two	Grade Three	Grade Four	Grade Five	Grade Six
Male	Excellent	100	1700	2000	2300	2600	2900	3200
		95	1600	1900	2200	2500	2800	3100
		90	1500	1800	2100	2400	2700	3000
	Good	85	1400	1650	1900	2150	2450	2750
		80	1300	1500	1700	1900	2200	2500
	Pass	78	1240	1430	1620	1820	2110	2400
		76	1180	1360	1540	1740	2020	2300
		74	1120	1290	1460	1660	1930	2200
		72	1060	1220	1380	1580	1840	2100
		70	1000	1150	1300	1500	1750	2000
		68	940	1080	1220	1420	1660	1900
		66	880	1010	1140	1340	1570	1800
		64	820	940	1060	1260	1480	1700
		62	760	870	980	1180	1390	1600
		60	700	800	900	1100	1300	1500
	Fail	50	660	750	840	1030	1220	1410
		40	620	700	780	960	1140	1320
		30	580	650	720	890	1060	1230
		20	540	600	660	820	980	1140
		10	500	550	600	750	900	1050
Female	Excellent	100	1400	1600	1800	2000	2250	2500
		95	1300	1500	1700	1900	2150	2400
		90	1200	1400	1600	1800	2050	2300
	Good	85	1100	1300	1500	1700	1950	2200
		80	1000	1200	1400	1600	1850	2100
	Pass	78	960	1150	1340	1530	1770	2010
		76	920	1100	1280	1460	1690	1920
		74	880	1050	1220	1390	1610	1830
		72	840	1000	1160	1320	1530	1740
		70	800	950	1100	1250	1450	1650
		68	760	900	1040	1180	1370	1560
		66	720	850	980	1110	1290	1470
		64	680	800	920	1040	1210	1380
		62	640	750	860	970	1130	1290
		60	600	700	800	900	1050	1200
	Fail	50	580	680	780	880	1020	1170
		40	560	660	760	860	990	1140
		30	540	640	740	840	960	1110
		20	520	620	720	820	930	1080
		10	500	600	700	800	900	1050

Table 3 Scoring criteria for the 50-meter run(s)

Sex	Rating	Score	Grade One	Grade Two	Grade Three	Grade Four	Grade Five	Grade Six
Male	Excellent	100	10.2	9.6	9.1	8.7	8.4	8.2
		95	10.3	9.7	9.2	8.8	8.5	8.3
		90	10.4	9.8	9.3	8.9	8.6	8.4
	Good	85	10.5	9.9	9.4	9.0	8.7	8.5
		80	10.6	10.0	9.5	9.1	8.8	8.6
	Pass	78	10.8	10.2	9.7	9.3	9.0	8.8
		76	11.0	10.4	9.9	9.5	9.2	9.0
		74	11.2	10.6	10.1	9.7	9.4	9.2
		72	11.4	10.8	10.3	9.9	9.6	9.4
		70	11.6	11.0	10.5	10.1	9.8	9.6
		68	11.8	11.2	10.7	10.3	10.0	9.8
		66	12.0	11.4	10.9	10.5	10.2	10.0
		64	12.2	11.6	11.1	10.7	10.4	10.2
		62	12.4	11.8	11.3	10.9	10.6	10.4
		60	12.6	12.0	11.5	11.1	10.8	10.6
	Fail	50	12.8	12.2	11.7	11.3	11.0	10.8
		40	13.0	12.4	11.9	11.5	11.2	11.0
		30	13.2	12.6	12.1	11.7	11.4	11.2
		20	13.4	12.8	12.3	11.9	11.6	11.4
		10	13.6	13.0	12.5	12.1	11.8	11.6
Female	Excellent	100	11.0	10.0	9.2	8.7	8.3	8.2
		95	11.1	10.1	9.3	8.8	8.4	8.3
		90	11.2	10.2	9.4	8.9	8.5	8.4
	Good	85	11.5	10.5	9.7	9.2	8.8	8.7
		80	11.8	10.8	10.0	9.5	9.1	9.0
	Pass	78	12.0	11.0	10.2	9.7	9.3	9.2
		76	12.2	11.2	10.4	9.9	9.5	9.4
		74	12.4	11.4	10.6	10.1	9.7	9.6
		72	12.6	11.6	10.8	10.3	9.9	9.8
		70	12.8	11.8	11.0	10.5	10.1	10.0
		68	13.0	12.0	11.2	10.7	10.3	10.2
		66	13.2	12.2	11.4	10.9	10.5	10.4
		64	13.4	12.4	11.6	11.1	10.7	10.6
		62	13.6	12.6	11.8	11.3	10.9	10.8
		60	13.8	12.8	12.0	11.5	11.1	11.0
	Fail	50	14.0	13.0	12.2	11.7	11.3	11.2
		40	14.2	13.2	12.4	11.9	11.5	11.4
		30	14.4	13.4	12.6	12.1	11.7	11.6
		20	14.6	13.6	12.8	12.3	11.9	11.8
		10	14.8	13.8	13.0	12.5	12.1	12.0

Table 4 Scoring criteria for sit-and-reach(cm)

Sex	Rating	Score	Grade One	Grade Two	Grade Three	Grade Four	Grade Five	Grade Six
Male	Excellent	100	16.1	16.2	16.3	16.4	16.5	16.6
		95	14.6	14.7	14.9	15.0	15.2	15.3
		90	13.0	13.2	13.4	13.6	13.8	14.0
	Good	85	12.0	11.9	11.8	11.7	11.6	11.5
		80	11.0	10.6	10.2	9.8	9.4	9.0
	Pass	78	9.9	9.5	9.1	8.6	8.2	7.7
		76	8.8	8.4	8.0	7.4	7.0	6.4
		74	7.7	7.3	6.9	6.2	5.8	5.1
		72	6.6	6.2	5.8	5.0	4.6	3.8
		70	5.5	5.1	4.7	3.8	3.4	2.5
		68	4.4	4.0	3.6	2.6	2.2	1.2
		66	3.3	2.9	2.5	1.4	1.0	-0.1
		64	2.2	1.8	1.4	0.2	-0.2	-1.4
		62	1.1	0.7	0.3	-1.0	-1.4	-2.7
		60	0.0	-0.4	-0.8	-2.2	-2.6	-4.0
	Fail	50	-0.8	-1.2	-1.6	-3.2	-3.6	-5.0
		40	-1.6	-2.0	-2.4	-4.2	-4.6	-6.0
		30	-2.4	-2.8	-3.2	-5.2	-5.6	-7.0
		20	-3.2	-3.6	-4.0	-6.2	-6.6	-8.0
		10	-4.0	-4.4	-4.8	-7.2	-7.6	-9.0
Female	Excellent	100	18.6	18.9	19.2	19.5	19.8	19.9
		95	17.3	17.6	17.9	18.1	18.5	18.7
		90	16.0	16.3	16.6	16.9	17.2	17.5
	Good	85	14.7	14.8	14.9	15.0	15.1	15.2
		80	13.4	13.3	13.2	13.1	13.0	12.9
	Pass	78	12.3	12.2	12.1	12.0	11.9	11.8
		76	11.2	11.1	11.0	10.9	10.8	10.7
		74	10.1	10.0	9.9	9.8	9.7	9.6
		72	9.0	8.9	8.8	8.7	8.6	8.5
		70	7.9	7.8	7.7	7.6	7.5	7.4
		68	6.8	6.7	6.6	6.5	6.4	6.3
		66	5.7	5.6	5.5	5.4	5.3	5.2
		64	4.6	4.5	4.4	4.3	4.2	4.1
		62	3.5	3.4	3.3	3.2	3.1	3.0
		60	2.4	2.3	2.2	2.1	2.0	1.9
	Fail	50	1.6	1.5	1.4	1.3	1.2	1.1
		40	0.8	0.7	0.6	0.5	0.4	0.3
		30	0.0	-0.1	-0.2	-0.3	-0.4	-0.5
		20	-0.8	-0.9	-1.0	-1.1	-1.2	-1.3
		10	-1.6	-1.7	-1.8	-1.9	-2.0	-2.1

Table 5 Scoring criteria for the 1-minute rope skipping

Sex	Rating	Score	Grade One	Grade Two	Grade Three	Grade Four	Grade Five	Grade Six
Male	Excellent	100	109	117	126	137	148	157
		95	104	112	121	132	143	152
		90	99	107	116	127	138	147
	Good	85	93	101	110	121	132	141
		80	87	95	104	115	126	135
	Pass	78	80	88	97	108	119	128
		76	73	81	90	101	112	121
		74	66	74	83	94	105	114
		72	59	67	76	87	98	107
		70	52	60	69	80	91	100
		68	45	53	62	73	84	93
		66	38	46	55	66	77	86
		64	31	39	48	59	70	79
		62	24	32	41	52	63	72
		60	17	25	34	45	56	65
	Fail	50	14	22	31	42	53	62
		40	11	19	28	39	50	59
		30	8	16	25	36	47	56
		20	5	13	22	33	44	53
		10	2	10	19	30	41	50
Female	Excellent	100	117	127	139	149	158	166
		95	110	120	132	142	151	159
		90	103	113	125	135	144	152
	Good	85	95	105	117	127	136	144
		80	87	97	109	119	128	136
	Pass	78	80	90	102	112	121	129
		76	73	83	95	105	114	122
		74	66	76	88	98	107	115
		72	59	69	81	91	100	108
		70	52	62	74	84	93	101
		68	45	55	67	77	86	94
		66	38	48	60	70	79	87
		64	31	41	53	63	72	80
		62	24	34	46	56	65	73
		60	17	27	39	49	58	66
	Fail	50	14	24	36	46	55	63
		40	11	21	33	43	52	60
		30	8	18	30	40	49	57
		20	5	15	27	37	46	54
		10	2	12	24	34	43	51

Table 6 Scoring criteria for the 1-minute sit-ups

Sex	Rating	Score	Grade Three	Grade Four	Grade Five	Grade Six
Male	Excellent	100	48	49	50	51
		95	45	46	47	48
		90	42	43	44	45
	Good	85	39	40	41	42
		80	36	37	38	39
	Pass	78	34	35	36	37
		76	32	33	34	35
		74	30	31	32	33
		72	28	29	30	31
		70	26	27	28	29
		68	24	25	26	27
		66	22	23	24	25
		64	20	21	22	23
		62	18	19	20	21
		60	16	17	18	19
	Fail	50	14	15	16	17
		40	12	13	14	15
		30	10	11	12	13
		20	8	9	10	11
		10	6	7	8	9
Female	Excellent	100	46	47	48	49
		95	44	45	46	47
		90	42	43	44	45
	Good	85	39	40	41	42
		80	36	37	38	39
	Pass	78	34	35	36	37
		76	32	33	34	35
		74	30	31	32	33
		72	28	29	30	31
		70	26	27	28	29
		68	24	25	26	27
		66	22	23	24	25
		64	20	21	22	23
		62	18	19	20	21
		60	16	17	18	19
	Fail	50	14	15	16	17
		40	12	13	14	15
		30	10	11	12	13
		20	8	9	10	11
		10	6	7	8	9

Table 7 Scoring criteria for the 50m×8 shuttle run(minutes・seconds)

Sex	Rating	Score	Grade Five	Grade Six
Male	Excellent	100	1'36"	1'30"
		95	1'39"	1'33"
		90	1'42"	1'36"
	Good	85	1'45"	1'39"
		80	1'48"	1'42"
	Pass	78	1'51"	1'45"
		76	1'54"	1'48"
		74	1'57"	1'51"
		72	2'00"	1'54"
		70	2'03"	1'57"
		68	2'06"	2'00"
		66	2'09"	2'03"
		64	2'12"	2'06"
		62	2'15"	2'09"
		60	2'18"	2'12"
	Fail	50	2'22"	2'16"
		40	2'26"	2'20"
		30	2'30"	2'24"
		20	2'34"	2'28"
		10	2'38"	2'32"
Female	Excellent	100	1'41"	1'37"
		95	1'44"	1'40"
		90	1'47"	1'43"
	Good	85	1'50"	1'46"
		80	1'53"	1'49"
	Pass	78	1'56"	1'52"
		76	1'59"	1'55"
		74	2'02"	1'58"
		72	2'05"	2'01"
		70	2'08"	2'04"
		68	2'11"	2'07"
		66	2'14"	2'10"
		64	2'17"	2'13"
		62	2'20"	2'16"
		60	2'23"	2'19"
	Fail	50	2'27"	2'23"
		40	2'31"	2'27"
		30	2'35"	2'31"
		20	2'39"	2'35"
		10	2'43"	2'39"
