# Supplementary material for: Associations of the Xingfa gamification framework with students' sport motivation and physical fitness: a cross-sectional comparison across 14 schools
Source: Front Psychol. 2026 Aug 27;17:1904485. doi: 10.3389/fpsyg.2026.1904485 (PMC13558109; doi:10.3389/fpsyg.2026.1904485)
Supplement: Supplementary file 2 [file Supplementary_file_2.pdf]

Supplementary Material A:

XFTPE Teacher Training Curriculum (30-Day Program)

Target Audience: Physical Education teachers from the 7 experimental schools.

Training Objective: To equip teachers with the theoretical knowledge of Self-Determination Theory (SDT) and the practical skills required to implement the structured, three-stage XFTPE model in natural settings.

Training Structure: The 30-day program is divided into four intensive modules, combining theoretical seminars, and field simulations.

Phase	Content & Objectives
Theoretical Foundations: SDT & Motivation<br>(Days 1-5)	**Content:**<br>(a) Introduction to Self-Determination Theory (SDT);<br>(b) Understanding “Needs Gap” in traditional PE: Why students dislike games followed by rigid drills;<br>**Objectives:**<br>(a) Teachers can articulate the three basic psychological needs.<br>(b) Teachers understand the mechanism of motivation internalization.
The Xingfa Framework: Methodology & Design<br>(Days 6-15)	**Content:**<br>(a) The Three-Stage Progression Logic:<br>Initial Xing (Autonomy): Designing low-threshold warm-ups in natural settings;<br>Middle Xing (Competence): Structuring skill practice with moderate gamified challenges;<br>Final Xing (Relatedness): Creating team-based application activities.<br>(b) Nature Integration: Utilizing school gardens, playgrounds, and outdoor spaces to reduce perceived control.<br>**Objectives:**<br>(a) Mastery of the 45-minute time allocation (10’/20’/15’);<br>(b) Ability to design activities that progressively satisfy psychological needs.
Practical Skill Application & Simulation<br>(Days 16-25)	**Content:**<br>(a) Micro-Teaching: Teachers deliver mock lessons implementing specific XFTPE stages.<br>(b) Peer Feedback: Analyzing peer lessons based on SDT principles.<br>(c) Gamification Techniques: Beyond rewards—using curiosity, challenge, and cooperation as drivers.<br>(d) Safety Management: Risk assessment for outdoor and nature-based activities.

	**Objectives:**<br>(a) Practical competence in delivering non-controlling instruction.<br>(b) Ensuring student safety during unstructured or semi-structured outdoor play.
Assessment, Fidelity, & Field Preparation<br>Days 26-30	**Content:**<br>(a) Assessment Tools: Training on using the *Sport Motivation Scale (SMS-6)* and interpreting student feedback.<br>(b) Fidelity Monitoring: Instructions on completing weekly checklists, implementation logs, and peer observation protocols.<br>(c) Resource Distribution: Handing out the *XFTPE Activity Handbook* and standardized lesson plans.<br>(d) Final Assessment: Teachers must design and present a complete XFTPE lesson plan for Grades 4-6.<br>**Objectives:**<br>(a) Ensure standardization across all 7 participating schools.<br>(b) Verify teacher readiness for the semester-long intervention.

Supplementary Material B:

Sample XFTPE Lesson Plan (Grade 6 - Basketball)

Lesson Topic: Ball Handling (Dribbling) and Teamwork
Target Group: Grade 6 Students
Duration: 45 Minutes
Location: Outdoor Basketball Court / Open Area
Equipment: Basketballs (1 per student), cones, colored vests.

1. Learning Objectives

1.1 Knowledge & Skills:

(1) Students will be able to control a basketball using their fingers and wrists for both high and low dribbles.

(2) Students will be able to change direction and speed while dribbling in response to instructions.

(3) Students will understand and execute basic teamwork principles (e.g., passing, movement off the ball).

1.2 Process & Methods:

(1) Students will experience the complete learning process from autonomous exploration to skill mastery and then to team application through gamified challenges.

(2) Students will learn to select appropriate practice difficulty based on their own abilities, fostering self-regulation and a willingness to challenge themselves.

1.3 Affective & Values:

(1) Students will experience the joy of movement in a non-structured game, enhancing their intrinsic interest in basketball.

(2) Students will learn to encourage and cooperate with peers, feeling a sense of collective achievement and belonging.

2. Student Analysis

2.1 Cognitive Characteristics: Sixth-grade students possess a certain level of abstract thinking and can understand and follow game rules. They have a high interest in competitive and cooperative activities and are eager to showcase themselves within a team.

2.2 Skill Foundation: Students have a preliminary grasp of stationary dribbling, but their coordination, ball control, and ability to combine dribbling with movement vary. Some students may find dribbling boring or frustrating.

2.3 Psychological Needs: Students of this age crave autonomy and peer recognition. They need successful experiences to build confidence and also require team activities to establish social connections.

3. Teaching Focus & Difficulties

3.1 Focus:

(1) The feel and control of the hand on the ball during dribbling.

(2) Applying dribbling skills in dynamic team games.

3.2 Difficulties:

(1) Maintaining control of the ball while moving, especially during defense and quick changes of direction.

(2) How to naturally use dribbling and passing skills in team games, rather than just focusing on individual scoring.

4. Teaching Process

Stage	Initial Xing (Desire Arousal)	Middle Xing (Skill Internalization)	Final Xing (Application in Nature)
Time	10 mins	20 mins	15 mins
Activity	Dribble Tag in the Wild: Each student dribbles a ball freely in a large outdoor zone. Students must try to tap another student's ball away while protecting their own. There are no strict rules on which hand to use or where to move.	Skill Gates Challenge: Students dribble through a series of "gates" (cones) set at varying distances. They can choose "Easy," "Medium," or "Hard" paths based on their confidence level.	Number Game: Two teams play on the court. The coach calls out a number (e.g., "3!"), and 3 players from each team run onto the court to play a 3v3 mini-game, attempting to score. All other players surround the court as active supporters/cheerleaders.
Implementation Details	Teacher Role: Monitor safety. Encourages creativity (e.g., "Try moving backwards or sideways!"). Environment: Uses the full outdoor space to minimize spatial constraints. Goal: Lower the threshold for participation; make students feel the ball is an extension of play, not a tool for rigid drills.	Teacher Role: Provides specific technical feedback (e.g., finger pads, knee bend). Differentiation: Allowing students to choose their difficulty level ensures they experience success and mastery. Goal: Enhance dribbling technique through gamified challenges that match individual skill levels.	Teacher Role: Facilitates fair play and highlights moments of good passing or teamwork. Social Aspect: The game encourages peer reliance and collective celebration of success. Goal: Apply the dribbling skill in a dynamic, socially supportive game context.

5. Teaching Evaluation

5.1 Formative Assessment

(1) Teacher Observation: Observe if students dare to try different dribbling styles during "Dribble Tag" and how well they master the technique in "Skill Gates"

based on their chosen difficulty.

(2) Peer Feedback: During the “Number Game,” encourage off-court students to cheer for their teammates and observe the team collaboration atmosphere.

5.2 Summative Assessment

(1) Skill Demonstration: After class, randomly select a few students to demonstrate their dribbling skills to check their technical proficiency.

(2) Self-Assessment: Ask students: “Which part of today’s class did you enjoy the most? Why?” to understand changes in their intrinsic motivation.

5.3 Evaluation Criteria

(1) Technical Mastery: The height and rhythm of the dribble should be stable.

(2) Engagement: Active participation in each segment, especially in team games.

(3) Cooperative Spirit: Willingness to communicate and cooperate with teammates.

Supplementary Material C:

Xingfa PE Activity Manual (Grade 6 - Basketball)

Title: Xingfa Framework for Physical Education (XFTPE) - Teacher Activity Manual
Version: 1.0
Target Audience: Physical Education Teachers in Experimental Schools
Purpose: To provide a comprehensive, theory-driven guide for implementing the Xingfa Framework, detailing activities and pedagogical strategies for each of the four core scenarios: Initial Xing, Middle Xing, Final Xing, and Comprehensive Literacy Assessment, with a strong emphasis on natural environments.

Introduction

Welcome to the Xingfa PE Activity Manual. This guide is designed to help you implement the Xingfa Framework, a gamification model based on Self-Determination Theory (SDT). The manual provides activity examples and pedagogical strategies for each of the four core scenarios: Initial Xing (Desire Arousal), Middle Xing (Skill Internalization), Final Xing (Extension & Transfer), and Comprehensive Literacy Assessment. Each scenario is designed to be conducted in natural settings to foster student engagement, internalized motivation, and holistic development.

Scenario 1: Initial Xing (Desire Arousal)

Core Task: To precisely capture and coordinate students' latent movement desires, providing a basis for personalized activity plans.

Pedagogical Principle: Cultivate intrinsic motivation by awakening students' original impulses through cultural immersion and embodied imagination in natural, low-load, and high-interest environments.

Activity Manual:

**Activity**	**Teacher Role**	**Natural Setting**
Nature-Based Strategy Games:<br>Students play strategy games like "Capture the Flag" or "Nature Scavenger Hunt" that require planning and teamwork, directly triggering their desire for challenge and knowledge.	Facilitator. Encourage creative problem-solving and strategic thinking.	School garden, forested area, large field
Traditional Folk Games:<br>Students play traditional games like "Chinese Chess" or "Go" on large outdoor boards, or card games in a natural setting.	Observer. Guide reflection on the strategic and cultural aspects of the games.	Playground, shaded outdoor area
Immersive Folk Dance & Music:<br>Students learn and perform traditional dances (e.g., Dragon Dance, Lion Dance) or play folk instruments in a natural, open space. The cultural atmosphere and	Instructor. Connect the activity to cultural heritage and community.	School courtyard, outdoor stage

physical movement foster a sense of identity and belonging.		
Seasonal Nature Exploration: Students explore the natural environment to observe seasonal changes, collect natural materials, and engage in related physical activities (e.g., kite flying, ice sliding in winter). This connects physical activity to an understanding of life cycles and health principles.	Guide. Facilitate discussions on the connection between nature, health, and culture.	School garden, local park, nature trail

Scenario 2: Middle Xing (Skill Internalization)

Core Task: To deconstruct the traditional "command-imitation" controlled classroom and transform the teacher's role into a guide and scene designer.

Pedagogical Principle: Use natural and game-based elements to reshape basic and specialized sports courses into exploration fields, focusing on skill automation and tactical understanding.

Activity Manual:

**Activity**	**Teacher Role**	**Natural Setting**
Obstacle Course Challenge: Dull physical training is gamified into a challenge experience using natural and simple equipment (logs, rocks, ropes). Students navigate the course, developing agility, balance, and coordination.	Coach. Provide targeted feedback on technique and form.	Grassy field, wooded area
Tactical Field Games: For sports like basketball or soccer, the game is played on a larger, natural field. The open space allows for more complex, unstructured play, encouraging students to develop their own strategies and tactics.	Facilitator. Guide tactical discussions and problem-solving.	Large outdoor field, park
Natural Terrain Training: For activities like running or hiking, the varied terrain (hills, trails) provides a natural challenge that enhances endurance and adaptability, making the training more engaging than a flat track.	Instructor. Emphasize effort and perseverance.	Hilly terrain, nature trail

Unique Feedback Mechanism: The teacher provides direct, in-the-moment feedback. For example, when a student struggles with a balance task on a log, the teacher offers specific cues. When a student successfully completes a challenging part of the course, the teacher highlights their effort and success, helping them perceive their progress.

Scenario 3: Final Xing (Extension & Transfer)

Core Task: To build an educational field centered on emotional connection and goal extension, transforming transient classroom “Xinghui” into lasting habits and intrinsic drive.

Pedagogical Principle: Leverage health sports and interdisciplinary sports courses to achieve deep transfer from physical actions to mental models, using the natural world as a classroom.

Activity Manual:

**Activity**	**Teacher Role**	**Natural Setting**
Outdoor First Aid & Survival Skills: Students practice basic first aid and survival skills (e.g., building a shelter, finding water) in a natural setting, fostering a respect for life and the development of practical, life-saving habits.	Guide. Facilitate reflection on life’s fragility and resilience.	Forested area, campsite
“Five Grains & Four Seasons” Course: Integrates physical activity with lessons on nature, culture, and health. For example, students might plant and harvest vegetables, or learn about traditional health practices related to the seasons.	Instructor. Connect physical activity to philosophical wisdom and cultural knowledge.	School garden, local farm
Community & Family Connection: Organize events where students can share their learning with families and the community, such as a “Nature Festival” or a “Traditional Games Day”. This extends the learning from school to the home and community.	Facilitator. Promote family and community bonding.	School grounds, local community space

Scenario 4: Comprehensive Literacy Assessment

Core Task: To serve as a key touchpoint for promoting the qualitative change of intrinsic motivation, transforming external feedback into self-confirmation of life expansion.

Pedagogical Principle: View evaluation as a mechanism for confirming motivation internalization, following the internal logic of Self-Determination Theory to retrospectively confirm students’ autonomy, competence, and relatedness.

Assessment Framework:

Coordination of Desires (Autonomy): Teachers observe and assess students’ level of intrinsic motivation during activities, respecting individual differences in engagement and preference.

Enhancement of Interest (Competence): Teachers assess students’ immersion and mastery in basic and specialized sports exploration, solidifying the experience of

competence through observation and feedback.

Activation of Mind (Relatedness): The assessment focuses on higher-order motor cognition and socio-emotional skills. Teachers evaluate students' resilience, innovation, and collaboration during complex tasks, as well as their understanding of cultural and health concepts.

Outcome: This observational and holistic assessment system ultimately leads to the crystallization of students' life aspirations. The teacher's observations and feedback are used to dynamically optimize the next round of personalized activity plans, making the assessment itself a trigger for renewed "Xingfa."

Supplementary Material D:

Xingfa Framework Fidelity Checklist

Teacher: ___________________________

Date: ___________________________

Lesson Topic: ___________________________

Instructions: After completing a lesson, please check the box that best describes your adherence to the Xingfa Framework principles. This checklist helps ensure the integrity and consistency of the Xingfa intervention.

Stage	Fidelity Item	Yes	No
Initial Xing	1. The activity was conducted in a natural setting?	☐	☐
	2. The teacher's role was primarily a facilitator, encouraging creativity and free play, rather than giving direct instructions?	☐	☐
	3. The activity was low-structured and open-ended, allowing students to explore at their own pace?	☐	☐
	4. Students showed signs of engagement, curiosity, and spontaneous movement?	☐	☐
Middle Xing	5. The activity introduced a moderate challenge to satisfy the need for competence?	☐	☐
	6. The teacher provided specific, constructive feedback on technique and effort?	☐	☐
	7. The activity was gamified to make skill practice more engaging (e.g., as a game or challenge)?	☐	☐
	8. Students demonstrated a sense of mastery or progress during the activity?	☐	☐
Final Xing	9. The activity was a team-based or cooperative game conducted in a natural setting?	☐	☐
	10. The teacher facilitated fair play and highlighted moments of good teamwork and communication?	☐	☐
	11. The activity fostered a sense of belonging and peer connection?	☐	☐
	12. Students appeared to enjoy the social aspect of the team activity?	☐	☐
Overall Implementation	13. The lesson followed the approximate time allocation for each stage (10'/20'/15')?	☐	☐
	14. The overall lesson structure aligned with the "Play-Practice-Apply" logic of the Xingfa Framework?	☐	☐
	15. Any significant deviations from the planned Xingfa structure were documented?	☐	☐

Supplementary Material E:

Xingfa Framework Classroom Observation Protocol

Observer: ___________________________
Date of Observation: ___________________________
Teacher Observed: ___________________________
Lesson Topic: ___________________________
School: ___________________________

Instructions: Please observe the lesson and rate the teacher's adherence to the Xingfa Framework principles using the 4-point scale below. Provide specific examples in the "Notes" column to support your ratings.

1=Not Observed;
2=Partially Observed ;
3=Observed;
4=Consistently Observed

Dimension	Behavioral Indicators	Rating (1-4)	Notes
Autonomy Support (Initial Xing)	(a) Teacher acts as a facilitator, not a director.<br>(b) Provides open-ended choices and encourages student initiative.<br>(c) Minimizes controlling language and commands.<br>(d) Creates a low-pressure, non-judgmental environment.		
Competence Support (Middle Xing)	(a) Provides specific, constructive feedback on effort and technique.<br>(b) Offers challenges that are appropriately matched to student skill levels.<br>(c) Focuses on mastery and improvement rather than just performance.<br>(d) Celebrates student progress and effort.		
Relatedness Support (Final Xing)	(a) Structures activities to require cooperation and communication.<br>(b) Encourages positive peer interaction and teamwork.<br>(c) Highlights and praises moments of good sportsmanship and collaboration.<br>(d) Fosters a sense of community and belonging.		
Natural Setting Utilization	(a) The majority of the lesson is conducted in a natural or outdoor		

	environment.<br>(b) The natural setting is integrated into the activity design.<br>(c) The environment is used to reduce perceived control and enhance engagement.		
Overall Xingfa Structure	(a) Time allocation is roughly 10 minutes for Initial Xing, 20 minutes for Middle Xing, and 15 minutes for Final Xing.<br>(b) The three stages are clearly distinct and purposefully linked.		

Supplementary Material F:

Xingfa Framework Teacher Implementation Log

Example:

Date: 2026/08/06

Teacher: Miss Li

Week of Implementation: 7

School: Xingfa School

Grade/Class: Grade 6

Lesson Topic: Basketball Dribbling

Activity Description:

1. Initial Xing (10 mins): "Dribble Tag in the Wild" on the outdoor court.
2. Middle Xing (20 mins): "Skill Gates Challenge" with cones of varying difficulty.
3. Final Xing (15 mins): "Number Game" 3v3 mini-game.

Student Engagement & Reactions:

1. High energy and laughter during Dribble Tag.
2. Most students chose the "Medium" gate, showing good self-assessment.
3. Strong team spirit and cheering during the mini-game.

Challenges or Issues Encountered:

1. One student was hesitant to participate in the mini-game.
2. A few students struggled with the "Hard" gate.

Modifications Made for Next Time:

1. Next time, I will pair the hesitant student with a supportive partner.
2. I will offer more "Easy" gate options and provide more individual coaching.

Supplementary Material G:

Summary of Fidelity Monitoring Results

Study: Xingfa Framework for Physical Education (XFTPE) Intervention Study
Monitoring Period: 9/16/2024 to 11/25/2024

1. Overview of Monitoring Data

To ensure the integrity of the XFTPE intervention, a comprehensive fidelity monitoring system was implemented across all participating schools. Data were collected through three primary sources: Teacher Self-Checklists, Peer Classroom Observations, and Teacher Implementation Logs.

Total Lessons Scheduled: 200 lessons;
Total Lessons Observed: 40 lessons;
Total Logs Submitted: 182 logs.

2. Implementation Adherence and Compliance

Adherence was defined as the delivery of the three XFTPE stages (Initial, Middle, Final Xing) in the correct sequence and with the intended pedagogical focus (Autonomy, Competence, Relatedness).

Metric	Result	Interpretation
Structural Adherence Rate	96.5%	96.5% of the observed lessons followed the prescribed “Initial-Middle-Final” structure without omitting any stage.
Time Compliance Rate	92.0%	92% of observed lessons adhered to the recommended time allocation (10’/20’/15’), with minor variations (± 2 minutes) due to school bell schedules.
Teacher Compliance	High	Based on self-checklists, teachers reported consistently using natural settings and gamified strategies as prescribed.

3. Conclusion

The fidelity monitoring results demonstrate a high level of implementation fidelity, underscoring the successful translation of the Xingfa Framework from theory to practice. As an emerging pedagogical philosophy gaining widespread acceptance, Xingfa Teaching has been recognized and endorsed by numerous school principals who have subsequently unified its implementation across their campuses. This strong top-down administrative support ensured that the framework was delivered consistently and with high teacher compliance regarding its theoretical underpinnings (SDT). While the standardized application of the intervention provides a robust basis for evaluation, we acknowledge that uncontrolled external factors may also have influenced the outcomes; therefore, the observed improvements in student motivation and physical literacy should be interpreted as the combined result of the XFTPE intervention and the broader educational context.
